# Identification and characterization of variants and a novel 4 bp deletion in the regulatory region of *SIX6*, a risk factor for primary open‐angle glaucoma

**DOI:** 10.1002/mgg3.290

**Published:** 2017-04-27

**Authors:** Mohd Hussain Shah, Noemi Tabanera, Subbaiah Ramasamy Krishnadas, Manju R. Pillai, Paola Bovolenta, Periasamy Sundaresan

**Affiliations:** ^1^ Department of Molecular Genetics Aravind Medical Research Foundation Madurai India; ^2^ Centro de Biología Molecular Severo Ochoa CSIC‐UAM Madrid Spain; ^3^ CIBERER, ISCIII Madrid Spain; ^4^ Glaucoma Clinic Aravind Eye Hospital Madurai India

**Keywords:** Gene expression regulation, primary open‐angle glaucoma, *SIX6*, vertical cup to disc ratio, zebrafish

## Abstract

**Background:**

Primary open‐angle glaucoma (POAG) is a complex disease of multigenic inheritance and the most common subtype of glaucoma. *SIX6* encodes a transcription factor involved in retina, optic nerve, and pituitary development. Previous studies showed a genetic association between the *SIX6* locus and POAG, identifying risk alleles. Whether these alleles are present also in the south Indian population is unclear.

**Methods:**

To address this question, the *SIX6* gene and an already characterized and highly conserved *SIX6* enhancer (Ch14:60974427‐60974430) were sequenced in two south Indian cohorts, respectively, composed of 65/65 and 200/200 POAG cases/age‐matched controls. We next used Taqman‐based allelic discrimination assay to genotype a common variant (rs33912345: c.421A>C) and the rs1048372 SNP in two cohorts, respectively, composed of 557/387 and 590/448 POAG cases/age‐matched controls. An additional cohort of 153 POAG cases was subsequently recruited to assess the association of the rs33912345:c.421A>C and rs10483727 variants with more prominent changes in two POAG diagnostic parameters: retinal nerve fiber layer thickness and vertical cup/disc ratio, using spectral domain optical coherence tomography. The activity of the newly identified enhancer variants was assessed by transgenesis in zebrafish and *luciferase* assays.

**Results:**

We identified two known rare and two common variants in the *SIX6* locus and a novel 4 bp deletion in the analyzed enhancer. Contrary to previous studies, we could not establish a significant association between the rs10483727 and rs33912345:c.421A>C variants and PAOG in the south Indian ethnicity but patients carrying the corresponding C or T risk alleles exhibited a dose‐dependent reduction of the thickness of the retinal nerve fiber layer and a significant increase in the vertical cup/disc ratio. Transgenesis in zebrafish and *luciferase* assays demonstrated that the newly identified 4 bp deletion significantly reduced reporter expression in cells of the retinal ganglion and amacrine layers, where human *SIX6* is expressed.

**Conclusion:**

Altogether, our data further support the implication of *SIX6* variants as POAG risk factors and implicates *SIX6* haploinsufficiency in POAG pathogenesis.

## Introduction

Primary open‐angle glaucoma (POAG) is the most common type of glaucoma. POAG affects approximately 60.5 million people worldwide and it is predicted to increase up to 79.6 million by 2020 (Quigley and Broman 2006). POAG is a neurodegenerative disease, associated with loss of tissue in the rim of the optic disc, with a consequent increase in the size of the central portion of the optic disc, known as “cup”. These changes lead to retinal ganglion cell (RGC) loss, optic nerve degeneration, and loss of vision. POAG is highly heterogeneous and associated with various genetic risk factors (Fingert 2011). Many of these factors have been identified through Genome‐Wide Association Studies (GWAS) and include variants of genes with different functions such as *CAV1/CAV2* (OMIM 601047, 601048; Wiggs et al. 2011), *CDKN2B‐AS1* (613149; Nakano et al. 2012; Osman et al. 2012; Ramdas et al. 2011; Wiggs et al. 2012), *TMCC4* (*TMCO1* 614123; van Koolwijk et al. 2012), *ATOH7* (609875; Macgregor et al. 2010), *GAS7* (603127; van Koolwijk et al. 2012), *ABC1* (*ABCA1*;600046; Chen et al. 2014; Gharahkhani et al. 2014), *AFAP1* (608252*;* Gharahkhani et al. 2014), *GMDS* (602884; Gharahkhani et al. 2014), *CDG1* (*PMM2*; 601785; Chen et al. 2014), *FNDC3B* (611909; Hysi et al. 2014; Lu et al. 2013), *TGFBR3* (600742; Li et al. 2015), *TXNRD2* (600742; Bailey et al. 2016), *SCA2 (ATXN2* 601517; Bailey et al. 2016), *FKHL7* (*FOXC1* 601090; Bailey et al. 2016), and *OPTX2/DFNA23 (SIX1/SIX6;* 601205, 606326*;* Osman et al. 2012; Wiggs et al. 2012). However, the most consistent associations have been observed for the *CDKN2B‐AS1* region on chromosome 9p21, followed by that of the *SIX1/SIX6* locus (Abu‐Amero et al. 2015).

The association with the *SIX1/SIX6* locus is particularly interesting because *SIX* genes are known to have a highly conserved and important role during embryonic development (Cordoba et al. 2001). Both *SIX1* and *SIX6* belong to the family of *SIX/sine oculis* transcription factors with six members in mammals (*SIX1‐SIX6*). All members of the family are characterized by the presence of two highly conserved domains: a homeobox domain and a *SIX* domain (Kawakami et al. 2000; Cordoba et al. 2001; Kumar 2009). *SIX1* is expressed in several tissues, comprising the optic vesicles and the limb mesenchyme but it is poorly expressed in the eye. In contrast, studies in different species, including humans, have shown that *SIX6* is highly expressed in the developing retina, especially in RGC and amacrine cells, and in the pituitary (Gallardo et al. 1999; Li et al. 2002; Conte et al. 2010). Its genetic inactivation in mice causes hypoplasia of both tissues, associated with a strong reduction or absence of the optic nerves (Li et al. 2002). Knockdown of the two zebrafish paralogs, *six6a* and *six6b*, lead to a similar phenotype characterized by small eyes and optic nerve hypoplasia (Carnes et al. 2014; Iglesias et al. 2014). In humans, a large deletion of chromosome 14q22.3‐q23 including *SIX6* was associated with anophthalmia and pituitary defects and mutations in *SIX6* have been found in sporadic anophthalmia (Gallardo et al. 1999, 2004; Nolen et al. 2006; Aldahmesh et al. 2013), further supporting the importance of *SIX6* in ocular development and related diseases. Although still poorly understood, a number of studies have also addressed the characterization of the *Six6* regulatory region in fish and mouse, identifying highly conserved regulatory elements (Conte et al. 2010; Lee et al. 2012), including one that drives *Six6* expression in the retina (Conte et al. 2010).

The first hint of *SIX6* implication in POAG came from studies that associated variants in the *SIX1‐SIX6* locus with alterations of the optic nerve head, such as that measured by the vertical cup to disc ratio (VCDR) (Macgregor et al. 2010; Ramdas et al. 2010), a parameter used to diagnose and monitor clinical POAG progression. Thereafter, a GWAS identified a strong POAG association with a single‐nucleotide polymorphism located in the intergenic region between the *SIX1* and *SIX6* locus (rs10483757, or T allele) (Wiggs et al. 2012), which was confirmed in different ethnic cohorts (Fan et al. 2011; Ramdas et al. 2011; Osman et al. 2012; Kuo et al. 2015). Quantitative trait locus analysis of *SIX1‐SIX6* has also shown a significant association of the rs10483757 allele with a decreased thickness of the retinal nerve fiber layer (RNFL) in individuals of European decent (Kuo et al. 2015). Furthermore, clinical, genetic, and functional studies identified a significant association of POAG with an additional variant located in the *SIX6* coding region (rs33912345:c.421A>C). Patients homozygous for the rs33912345 risk allele (or the C risk allele) had a thinner RNFL than patients homozygous for the A nonrisk allele (Carnes et al. 2014; Cheng et al. 2014). Whether these risk alleles are common also in the south Indian population is still unclear.

To address this issue and determine a possible genetic association of the rs33912345:c.421A>C and rs10483727 (*SIX1‐SIX6*) SNPs, we sequenced the *SIX6* locus in a cohort of south Indian POAG patients and age‐matched controls and further compared the resulting genotypes with the RNFL and VCDR parameters. We identified already known common and rare variants. In addition, we discovered a novel 4 base pair (bp) deletion within the *SIX6* regulatory region in three POAG cases (Ch14:60974427‐60974430). Because this deletion fell in a characterized and highly conserved enhancer region that drives *Six6* expression in the vertebrate retina (Conte et al. 2010), we used transgenesis in zebrafish and luciferase assays to show that the presence of the deletion strongly reduces reporter expression, suggesting that low *SIX6* expression might be implicated in POAG pathogenesis.

## Materials and Methods

### Ethical compliance

The study adhered to the tenets of the Declaration of Helsinki, and ethics committee approval was obtained from the Institutional Review Board of the Aravind Eye Care System. All subjects read and signed informed consent except for illiterate subjects, who had the information leaflet read out and provided a thumb impression for participation consent. Experiments in zebrafish were performed according to the European regulations for animal work and received Institutional and governmental approval under the following authorization numbers: PROEX 100/15 and ES/12/I‐30.

### Patients

The subjects for this study (cases and controls) were recruited from the outpatient services of a tertiary eye care hospital in south India (Aravind Eye Care System, Madurai, India). Cases were individuals diagnosed for POAG as described below, and controls were individuals with normal intra‐ocular pressure (IOP), open‐angles, with no evidence of any ocular disease except senile cataracts or refractive errors ≤3 D spherical equivalent.

### Ocular examination

Study participants received a comprehensive eye examination. Best‐corrected visual acuity was obtained by refraction and ocular pressures were measured by the Goldmann applanation tonometer (Haag‐Striet, Switzerland) prior to pupil dilatation. IOP of each eye was recorded as the median of three readings. Central corneal thickness was determined as the mean of three consecutive measurements with an ultrasonic pachymeter. Slit‐lamp biomicroscopic evaluation of the anterior segment and pupils was followed by gonioscopic evaluation of the anterior chamber angle, using Goldman II Gonioscopic mirror. Static autoperimetry (SITA standard 24‐2, Humphrey Field Analyzer II, Zeiss) was performed prior to pupil dilation. The pupils were dilated using tropicamide 1% and the posterior pole was evaluated with a 90D aspheric lens to assess the optic nerve head and the peripapillary retinal nerve fiber layer. VCDR was measured with an eye‐piece measuring reticule.

### Optical coherence tomography

We selected a subset of 153 individuals affected by POAG, whose diagnosis was confirmed on the basis of their optic disc characteristics and matching visual field defects by Humphrey autoperimetry. These individuals were subjected to optic disc and RNFL evaluation using spectral–domain optical coherence tomography (SD OCT, Topcon 3D OCT 2000), after obtaining adequate view of the posterior pole of the eyes by pharmacologic mydriasis. Optic nerve head and RNFL scan acquisitions were performed for each one of the participants. The subjects’ pupil was centered and focused in an iris‐viewing camera on the acquisition screen and the lines scanning ophthalmoscope was used to obtain an optimized view of the retina in autofocus mode. For each one of the subjects, the optic nerve head and RNFL image acquisitions were obtained in 6 × 6 mm^2^ grid. The peripapillary measurement area was centered on the optic disc prior to the acquisition of the images. The rescanning function was automatically activated to compensate for any ocular movement, which could impede scanning of the appropriate area (motion correction compensation/rescanning function of the OCT). RNFL and ONH imaging scans were repeated several times if motion artifacts were detected. The ONH and RNFL algorithms were used to quantitatively estimate the optic disc area and peripapillary RNFL thickness (overall global average, quadrant wise average RNFL for each of the examined eyes of the subjects). Eyes with OCT scan with retinal layer segmentation errors, signal strength <7 or artifacts due to ocular movements were repeated to obtain reliable parameters. Those with recurrent retinal segmentation errors or artifacts were excluded from analysis.

### Definition of glaucoma

POAG definition included the presence of glaucomatous optic disc damage or neuropathy, matching visual field defects of nerve fiber bundle type on stati‐autoperimetry, normal open‐angle on gonioscopy, and absence of features suggestive of secondary glaucoma including exfoliation and pigment dispersion. Subjects with a VCDR of 0.6 or higher, or a narrowest neuroretinal rim width of <0.2 (including classic notching of neuroretinal rim) or VCDR asymmetry of 0.2 between the eyes coupled with visual field defect in a matching location in static autoperimetry were considered to have glaucomatous optic nerve damage or atrophy. The presence of significant optic disc excavation compatible with glaucoma (as above) or end‐stage glaucoma with significant central visual loss or total disc cupping was sufficient criteria for diagnosing glaucomatous optic nerve damage in individuals with unavailable visual fields due to poor visual acuity or inadequate reliability. Individuals with primary or secondary angle closure and all forms of secondary open‐angle glaucoma were excluded from this study.

### DNA sequencing

Genomic DNA was extracted from peripheral blood and the two exons, including exon/intron junctions, of the *SIX6* (NM_0007374.2;ENST00000327720.5) gene was amplified in 65/65 POAG cases/age‐matched controls using PCR with already tested primers (Carnes et al. 2014). The retinal *SIX6* enhancer element was amplified with the following primers (FW: 5′‐CGAGTGAACTGT GAAGATCTGTG‐3′; RV: 5‐AGGTGAGAACGTTCA CAGC CGA‐3′) in 200/200 POAG cases/age‐matched controls from the south Indian population. PCR products were purified and sequenced using BigDye chemistry in a 3730 DNA Analyzer (Applied Biosystems, Foster City, CA, USA).

### Genotyping

We genotyped a common variant (rs33912345: c.421A>C) and the rs1048372 SNP in two cohorts, respectively, composed of 557/387 and 590/448 POAG cases/age‐matched controls. Genotyping was performed using a real‐time based allelic discrimination Taqman SNP Assay (Applied Biosystems). Reactions were performed in 384‐well MicroAmp optical reaction plates using the ABI 7900 HT fast Real‐time thermo cycler. Data analysis was performed with the associated sequence detection systems Software version 2.3. Genotypes on the allelic discrimination plot with a quality value ≥96% and a Ct value between 21 and 30 were included in the results. The rs33912345 and rs1048372 alleles at *SIX1‐SIX6* locus were tested for association with POAG by logistic regression model adjusted for age and sex with PLINK 1.07 (Purcell et al. 2007).

### Statistical analysis

Data were analyzed with the PLINK 1.07 (Purcell et al. 2007) and STATA 12.1 software (StataCorp LP, College Station, TX, USA). Categorical variables were presented as frequency (percentage). Continuous variables were presented as mean ± SD. The association between cases and controls with individual alleles was evaluated with Chi‐square test. The possible relationship of the rs33912345 and rs10483727 variants with VCDR across the three genotypes was established with the Trend test. Logistic regression was used to calculate OR's with 95% confidence interval for allelic association test. Multiple regression analysis was used to explain the relationship between rs33912345 and rs10483727 with RNFL thickness. We assumed an additive generic model where the SNP dosage varies from 0, 1, or 2, representing the number of copies of carried risk allele (the C allele of rs33912345 and the T allele of rs10483727). Primary analysis for RNFL was adjusted for age, sex, and spherical equivalent. *P* value <0.05 was considered statistically significant.

### Cell transfection and luciferase assay

The identified SIX6 enhancer alleles were tested using luciferase reporter assay. A fragment of the reference and deleted enhancer were amplified from genomic DNA using specific primers (FW: 5′‐CGAGTGAACTGTGAAGATCTGTG ‐3′; RV: 5′‐AGGTGAGAACGTTCACAGCCGA‐3′) and cloned in the pGL3 plasmid containing a minimal promoter. HEK293 cells were plated in a 12‐well cluster plate and transfected 24 h after with the Lipofectamine‐2000 reagent, following the manufacturer's guidelines. Cells were transfected with the reporter (50 ng) and the pCMV‐*β*‐gal (10 ng) plasmid, used for normalization. Experiments were performed in triplicate and replicated at least three times as described (Conte et al. 2010; Beccari et al. 2015).

### Transgenesis in zebrafish

The retinal enhancer from the genomic DNA of a reference and a deleted POAG patient were amplified with the following primers: FW: 5′‐AGCAGCTAGGCGCTGG GATCGG‐3′ and RV: 5′‐TTCCAGTTGCCAAAGCAGCTGAG‐3′. The amplified fragments were cloned using the pCR8/GW/TOPO cloning kit (Invitrogen, CA, USA) and then transferred to the ZED vector (Bessa et al. 2009). Zebrafish embryos were microinjected at the one‐cell stage with 1–2 nL of a solution containing 40 nm of each construct and 50 ng/μL^−1^ of the *Tol2* mRNA. A minimum of 250 embryos per construct was injected. Putative transgenic embryos, as determined by the expression of the *cardiac‐actin:RFP* (internal transgenesis control), were screened for tissue‐specific enhancer activity. Positive embryos were grown to adulthood and three independent F1 lines were generated for each one of the constructs. Embryos derived from the F1 were then analyzed for GFP distribution in cryostat retina sections, immunoprocessed with anti‐GFP antibodies (Abcam, Cambridge, UK, raised in chick and diluted 1:2000 in PBT) followed by Alexa‐488‐conjugated secondary antibody (Life Technology, CA, USA). Sections were counterstained with Hoechst (Sigma, MO, USA) to visualize the nuclei.

## Results

### Screening and genotyping *SIX6* in a cohort of POAG and control individuals

**Figure 1:**
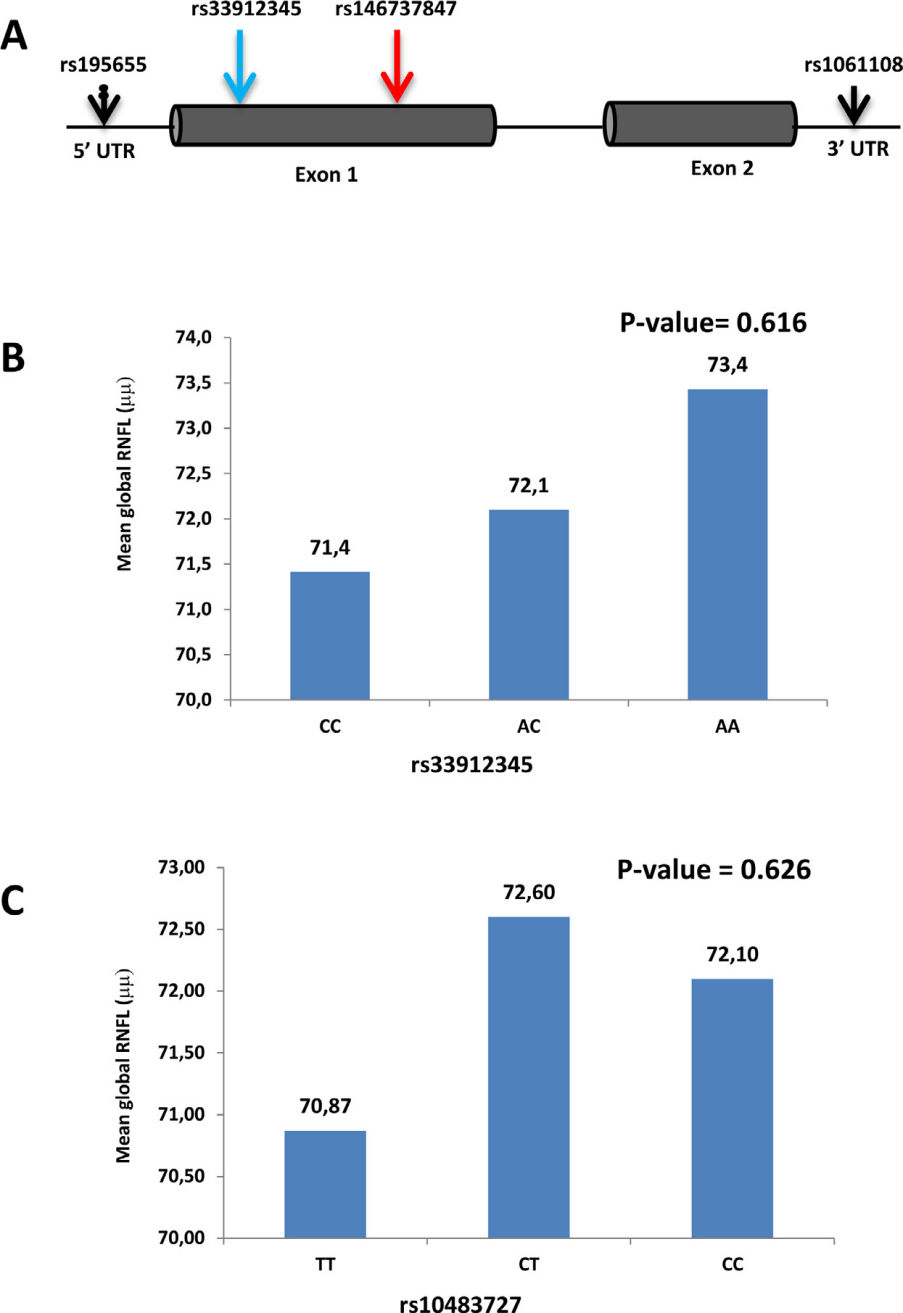
Localization of the SNPs identified in the *SIX6* gene and correlation of risk alleles with changes in RNFL thickness. (A) Schematic representation of the *SIX6* gene showing variants identified at coding and untranslated regions. (B, C) The graph shows the mean thickness of the RNFL among the three genotypes of the rs33912345 (B) and rs10483727 (C) variants in POAG cases. Patients with two copies of the C risk allele have a thinner global RNFL, whereas heterozygous genotypes have intermediate levels as compared with the nonrisk AA allele (B). Patients with two copies of the T risk allele presented a thinner global RNFL than those carrying a CT or CC nonrisk genotype (C). Data demonstrates genotype–phenotype correlation. Analysis was adjusted for age, sex, and SE.

We sequenced the *SIX6* gene in 65 POAG cases and 65 controls and identified four SNPs at different positions. These included two rare and two common variants, which were reported previously (Carnes et al. 2014) (Fig. 1A, Table 1). We then used Taqman‐based allelic discrimination assay to genotype one of the two common *SIX6* missense variants (rs33912345, Genebank: NM_007374.2, c.421A>C; Genebank: NP_031400.2, p.Asn141His) in 557 POAG cases and 387 age‐matched controls from south India, without finding an association between this variant and the disease (Table 2). A similar study for the rs1048372 SNP (located within *SIX1‐SIX6* locus) in 590 south Indian POAG cases and 448 age‐matched controls also failed to establish a significant association with the disease (Table 3), confirming the conclusions of a previous study (Philomenadin et al. 2015).

**Table 1:** Variants identified in the *SIX6* gene and its regulatory region

Gene	Coordinates^a^	SNP ID	# of cases/control	Phenotype	Function	Base change	AA^b^ change
*SIX6*	Chr14:60976537	rs33912345	130	Normal/POAG	Missense	A>C	Asn141His
*SIX6*	Chr14:60976501	rs146737847	130	POAG	Missense	G>A	Glu129Lys
*SIX6*	Chr.14:60975956	rs1956558	130	POAG	5′ UTR	T>C	‐
*SIX6*	Chr14:60978071	rs1061108	130	Normal/POAG	3′ UTR	G>C	‐
*SIX6*‐Enh	Chr14:60974427‐ 60974430	4 bp del	400	POAG^c^, 3 Control, 1	‐	del ATCT	‐

The variants and the 4 bp deletion were identified by sequencing POAG patients and age‐matched controls.

a Coordinates are based on the Hg19 reference.

b AA, amino acid.

c 4 bp deletion identified in three POAG patients and in one control.

**Table 2:** Association study of the rs33912345 variant with south Indian POAG

	POAG	Control
Number of samples (*n*)	557	387
Age [mean ± SD (range)]	60.6 ± 10.0 (24–86)	61.7 ± 7.1 (31–85)
Sex [*n* (%)]	Female ‐ 197 (35.4)	Female ‐ 200 (51.7)
	Male ‐ 360 (64.6)	Male ‐ 187 (48.3)

CHR	SNP	BP	A1	F_A	F_U	A2	CHISQ	*P*	OR	SE	L95	U95
14	rs33912345	60976537	A	0.3487	0.3681	C	0.7428	0.3888	0.919	0.09798	0.7584	1.114

The top part of the table shows the demographic features of cases and controls. The two bottom lines show the frequency of the rs33912345 allele among cases and control groups. *P* value comparison among the cases and control group was determined by *t*‐test. *P* values ≤0.05 were considered significant; value >0.05 indicates age‐matched group. Abbreviations: CHR, chromosome; SNP, SNP ID; BP, physical position (base pair); A1, minor allele name; FA, frequency of this allele in cases; FU, frequency of this allele in controls; A2, major allele name; CHISQ, basic allelic test chi‐square. Asymptotic *P* value for this test; OR, estimated Odds ratio; L95, Lower bound of 95% confidence interval for odds ratio; U95, Upper bound of 95% confidence interval for odds ratio.

**Table 3:** Association study of the rs10483727 variant with south Indian POAG

	POAG	Control
Number of samples (*n*)	590	448
Age [mean ± SD (range)]	59.7 ± 11.4 (24–85)	62.1 ± 7.0 (31–85)
Sex [*n* (%)]	Female ‐ 209 (35.4)	Female ‐ 232 (51.8)
	Male ‐ 381 (64.6)	Male ‐ 216 (48.2)

CHR	SNP	BP	A1	F_A	F_U	A2	CHISQ	*P*	OR	SE	L95	U95
14	rs10483727	60606157	C	0.3676	0.3792	T	0.2937	0.5879	0.9515	0.09167	0.7951	1.139

The top part of the table shows the demographic features of cases and controls. The two bottom lines show the frequency of the rs10483727 allele among cases and control groups. *P* value comparison among the cases and control group was determined by *t*‐test. *P* values ≤0.05 were considered significant; value >0.05 indicates age‐matched group. Abbreviations: CHR, chromosome; SNP, SNP ID; BP, physical position (base pair); A1, minor allele name; FA, frequency of this allele in cases; FU, frequency of this allele in controls; A2, major allele name; CHISQ, basic allelic test chi‐square; Asymptotic *P* value for this test; OR, estimated Odds ratio; L95, Lower bound of 95% confidence interval for odds ratio; U95, Upper bound of 95% confidence interval for odds ratio.

Since OCT images were not available in all the individuals of the cohorts described above, we next recruited 153 new POAG patients, who were characterized by glaucomatous optic neuropathy on ophthalmoscopic evaluation with matching visual field defects on autoperimetry. These individuals were analyzed with OCT to study RNFL and VCDR across the three genotypes of two SNPs (rs33912345:c.421A>C, rs10483727). Because age is known to influence retinal thickness, we took into account age at both POAG diagnosis and OCT measurement and compared only age‐matched individuals. Individuals carrying one rs33912345 “C” risk allele showed a decrease of −1.17 μm in RNFL thickness after adjusting for age, sex, and spherical equivalent (*P* = 0.554; Table 4), as compared with the POAG cases carrying the nonrisk allele. The strongest decrease was observed in the superior quadrant (−4.32 μm) followed by that in the temporal‐inferior one. Global RNFL was even thinner in subjects with C/C genotypes, compared to subjects with A/C and A/A genotypes (Fig. 1B). A similar analysis in individuals with the rs10483727 variant showed a decrease of −0.75 μm in the RNFL thickness (*P* = 0.708; Table 5). Indeed, after adjusting for age, sex, and spherical equivalent, there was a tendency to a thinner global RNFL in individuals with the T/T risk genotype, followed by C/C and C/T genotypes (Fig. 1C), although the data did not reach statistical significance. Also in this case, the strongest decrease in thickness was observed in the superior followed by temporal‐nasal inferior sectors.

**Table 4:** Association between the *SIX6* rs33912345 variant and reduction in the RNFL thickness

rs33912345_C (RAF = 0.68)	Model 1					Model 2				
	*β*	SE	*N*	*n*	*P* value	*β*	SE	*N*	*n*	*P* value
Nerve fiber thickness (Global)	−0.91324	1.846925	153	296	0.621	−1.17	1.98	121	219	0.554
Inferior	−2.09587	2.667079	153	302	0.432	−1.63	3.02	122	223	0.591
Superior	−4.8123	2.892626	153	303	0.096	−4.32	3.19	122	223	0.175
Nasal	1.315929	2.130075	153	300	0.537	2.24	2.33	121	221	0.337
Temporal	−2.03252	1.698793	153	300	0.232	−2.65	2.01	122	221	0.189

Measures were obtained with OCT. Analysis 1: data were normalized for age and gender. Analysis 2: data were normalized for age, gender, and SE. *β*: Changes in RNFL thickness (in μm) per copy of the “C” risk allele. RAF, risk allele frequency; *N*, number of individuals; *n*, number of eyes; SE, standard error.

**Table 5:** Association between the *SIX6* rs10483727 variant and reduction in the RNFL thickness

rs10483727_T (RAF = 0.65)	Analysis 1					Analysis 2				
	*β*	SE	*N*	*n*	*P* value	*β*	SE	*N*	*n*	*P* value
Nerve fiber thickness (Global)	−0.92267	1.872791	152	294	0.622	−0.75	2.01	121	218	0.708
Inferior	−1.86372	2.68792	153	302	0.488	−1.15	3.05	122	223	0.706
Superior	−4.738	2.91451	153	303	0.104	−3.78	3.22	122	223	0.239
Nasal	0.967713	2.142153	153	299	0.651	1.86	2.35	121	220	0.428
Temporal	−1.43413	1.716589	153	300	0.403	−1.97	2.04	122	221	0.335

Measures were obtained with OCT. Analysis 1: data were normalized for age and gender. Analysis 2: data were normalized for age, gender, and SE. *β*: Changes in RNFL thickness (in μm) per copy of the “T” risk allele. RAF, risk allele frequency; *N*, number of individuals; *n*, number of eyes; SE, standard error.

**Figure 2:**
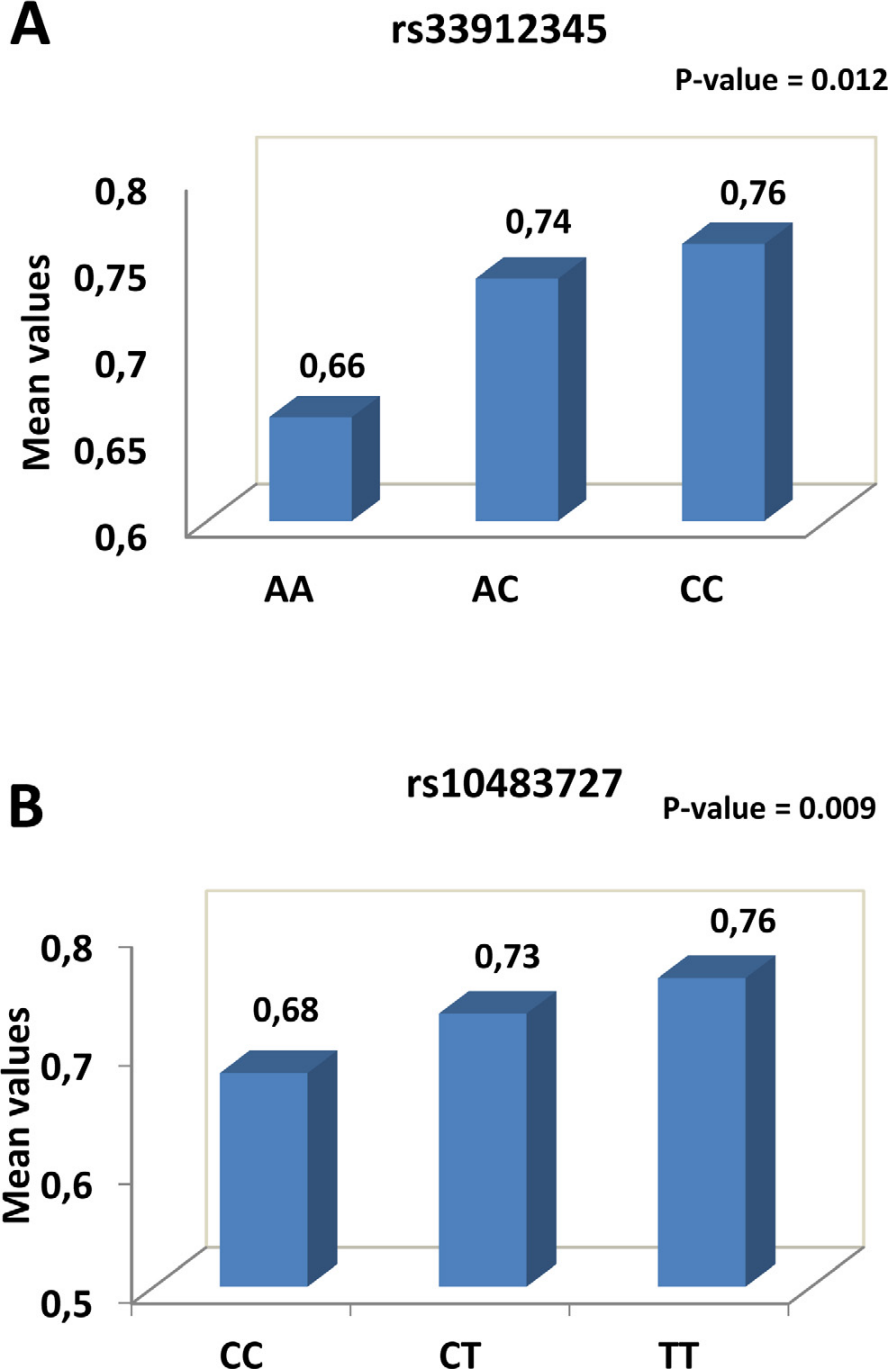
Correlation of two risk alleles with changes in the VCDR. (A, B) The graphs show the mean value of the vertical cup disc ratio (VCDR) in POAG cases homologous or heterozygous for (A) the A or C risk allele the rs33912345 variants and (B) the C or T risk allele of the rs10483727 variants in *SIX6*. Note that individuals carrying two copies of the “C” (A) or “T” (B) risk allele present a statistically significant higher value of the VCDR.

We next investigated rs33912345:c.421A>C and rs10483727 association with changes in VCDR. This ratio progressively increases in POAG individuals and is routinely used for glaucoma diagnosis (Ramdas et al. 2010). Supporting the association established with RNFL analysis, patients carrying two copies of the C or of the T risk allele presented a statistically significant increase in the VCDR value (*P* = 0.012; *P* = 0.009, respectively; Fig. 2A, B), as compared to POAG cases carrying the nonrisk alleles.

### Identification and functional analysis of novel 4 bp deletion in a *SIX6* enhancer

**Figure 3:**
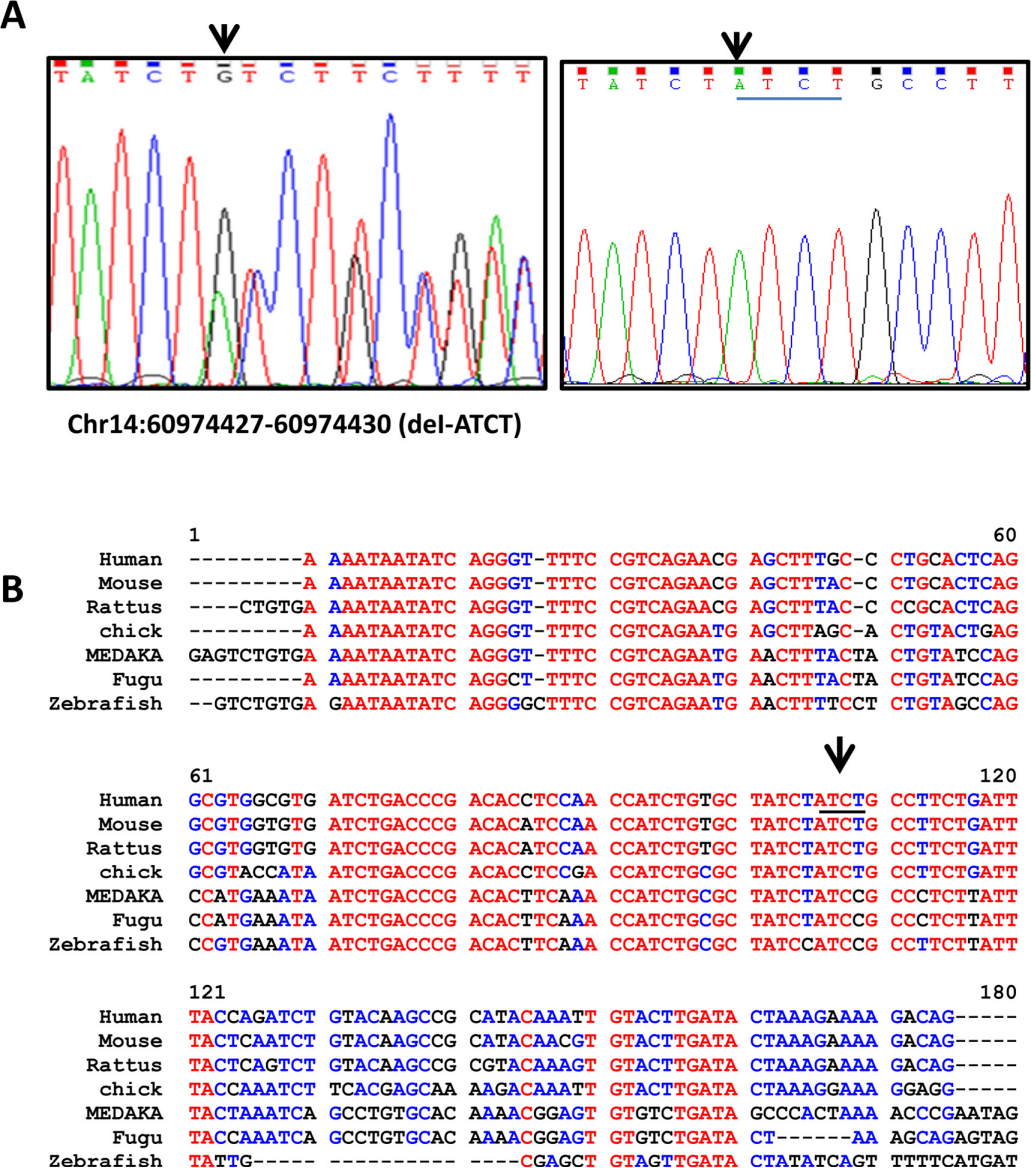
Identification of a 4 bp deletion in an evolutionary conserved *SIX*6 enhancer in POAG patients. (A) The relevant portions of the sequencing chromatogram of the PCR‐amplified genomic fragments of the *SIX6* enhancer (Chr14: 60974427‐60974430) illustrate the 4 bp deletion in a POAG patient (left, arrow indicates the position of the deletion) as compared to a reference individual (right, the deleted sequence is underlined). (B) Alignment of the genomic sequences of the retinal enhancer from different vertebrate species. Conserved nucleotides are indicated in red or blue, nonconserved in black. The position of the 4 bp deletion found in POAG patients is indicated by an arrow and underlined in the human sequence. Note that the deletion falls is a region of strong conservation.

Carnes et al. (Carnes et al. 2014) proposed that POAG risk could be associated not only to alterations in protein function but also to levels of gene expression. To determine if this could be the case in our cohort, we sequenced a retinal‐specific *SIX6* enhancer region in 200 POAG cases and 200 controls. Notably, we identified a novel 4 bp deletion (ATCT) in the enhancer region of the *SIX6* gene (Ch14:60974427‐60974430) in three POAG cases (Fig. 3A). This 4 bp deletion was present also in one age‐matched individual included in the control group (Table 1). This individual presented no glaucoma phenotype according to the clinical exam: his retinal nerve fiber layer was normal (0.2 VCDR) with no attributes typical of glaucomatous optic neuropathy. Notably however, he presented cataract, suffered from diabetes, and was born from a consanguineous marriage, suggesting that he might develop glaucoma in the future, as both diabetes and consanguinity are strong glaucoma risk factors (Chavez et al. 2013; Zhou et al. 2014). This individual has been scheduled for visual fields and OCT evaluation and he is under periodical clinical follow‐up to detect the development of possible characteristic POAG optic nerve damage.

**Figure 4:**
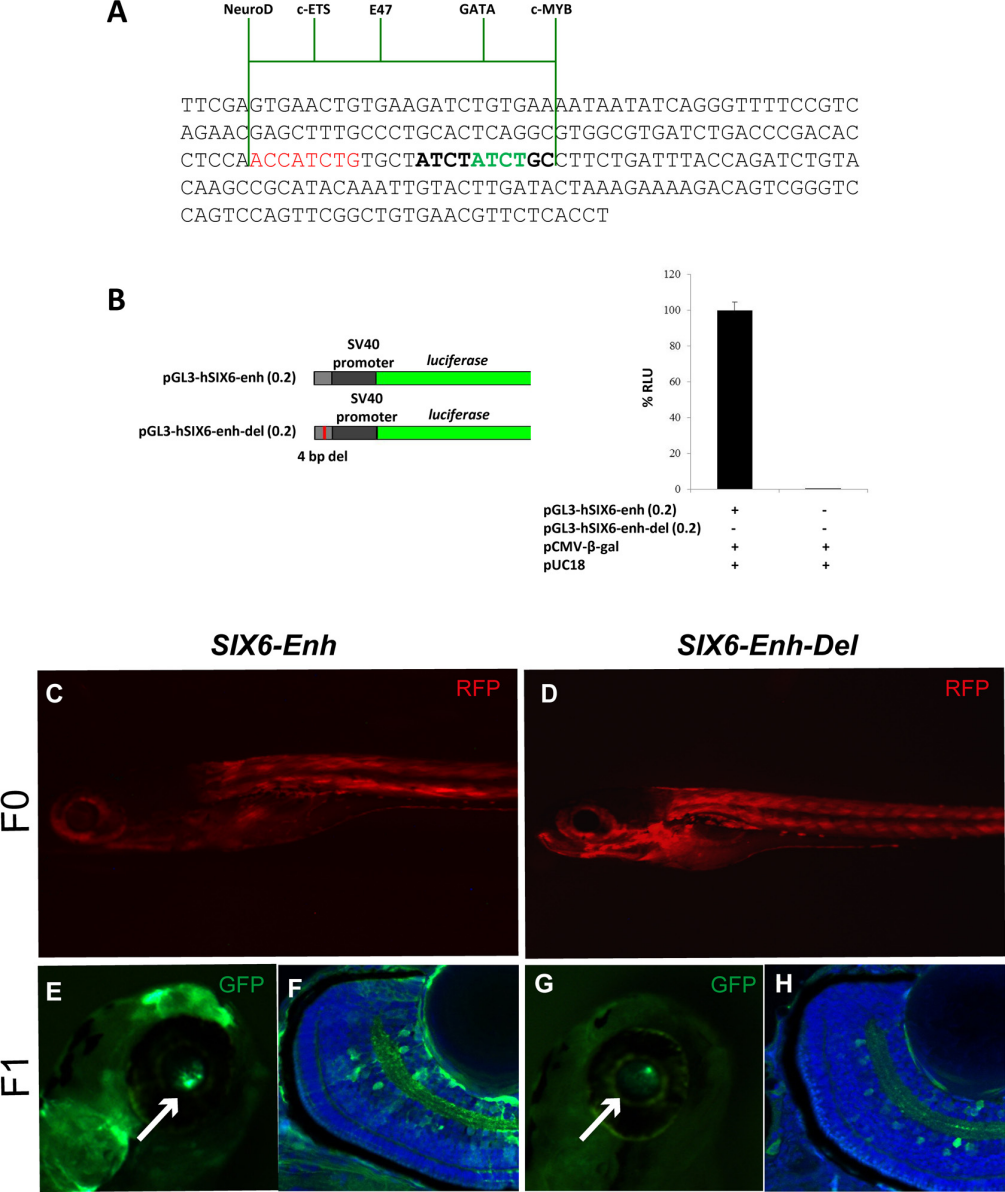
Functional characterization of the 4 bp deletion in the *SIX*6 enhancer found in POAG patients. (A) Sequence of the short enhancer fragment used to perform luciferase assays. The deleted sequence is indicated in green, the NeuroD‐binding site in red. The position of binding sites for the transcription factors ETS, E47, GATA, and C‐MYB is indicated with green lines. (B) Schematic representation of the constructs used to perform luciferase assays (left) and graph of the luciferase assay performed with the two different constructs and control plasmids as indicated in the panel. Bars represent % mean values ± SEM normalized to control; ***P < 0.0001. (C–H) Images of representative transgenic embryos generated with a human reference *SIX*6 retinal enhancer (C, E, F) and an enhancer containing the 4 bp deletion found in POAG patients (D, G, H). Embryos in (C, D) represent F0 transgenic embryos expressing control RFP at similar levels. (E–H) Images of representative F1 transgenic embryos showing GFP reporter expression driven by the reference (E, F) or deleted enhancer (G, H). Images in (e, g) are high power in toto view of the eye (arrows), whereas those in (F, H) are the corresponding frontal cryostat sections. Note how the reference enhancer drives strong GFP expression (green) in cells of the retinal ganglion and amacrine layers (F), whereas the deletion strongly downregulates reporter expression (H). Sections are counterstained with Hoechst (blue). Abbreviations: acl, amacrine cell layer; gcl, ganglion cell layer.

The identified 4 bp deletion fell within a previously characterized and evolutionarily conserved enhancer region (Fig. 3B), which has been shown to control *Six6* expression in the vertebrate retina (Conte et al. 2010). As the deleted sequence was reasonably conserved from fish to humans, we asked if the identified deletion could impair *SIX6* expression. To test this possibility, we first amplified a sequence of about 200 bp around the deleted region from the DNA of a corresponding patient as well as from that of a reference individual (Fig. 4A). We then compared the ability of the amplified fragments to drive *Luciferase* reporter expression in transfected HEK293 cells. While the region from a control individual activated reporter expression, that containing the 4 bp deletion had no activity (Fig. 4B), suggesting that the deleted 4 bp are relevant to the enhancer function.

To corroborate this possibility, we first inserted the entire control human enhancer sequence in the ZED transgenic vector (Bessa et al. 2009) and investigated if the plasmid could drive GFP reporter expression in the retina of zebrafish embryos, similarly to what has been previously observed for the medaka orthologous region (Conte et al. 2010). We generated 38 F0 transgenic embryos, as determined by RFP expression in the somites (Fig. 4C) that acts as a positive control (Bessa et al. 2009). About half of them (18) showed a clear GFP expression driven by the human enhancer element in the eye and occasionally in other CNS regions. We grew these embryos to adulthood and established three stable independent zebrafish lines. Analysis of the corresponding F1 (Fig. 4E), that we named Tg(hSix6_Enh:GFP)^cbm10^, indicated that the human SIX6 enhancer element consistently drove GFP expression specifically in a subpopulation of cells within the RGC and amacrine cell layers (Fig. 4F), where *SIX6* has been shown to be expressed in humans (Gallardo et al. 1999). Notably, similar experiments using the enhancer carrying the 4 bp deletion gave very different results. We established 56 F0 transgenic embryos (Fig. 4D) but only 10 of them (about 17%) showed a weak GFP expression in the eye. Analysis of the eye of the corresponding established F1 transgenic lines, named Tg(hSix6_Enh_del:GFP)^cbm11^, revealed weak or no GFP expression (Fig. 4G) and histological analysis of the retinas show poor or no signal in cells of the RGC and amacrine cell layers (Fig. 4H). This suggests that the 4 bp deletion impairs enhancer function. In support of this conclusion, we found that the sequence including the deletion and its immediate surroundings contains putative binding sites for different transcription factors, as determined with four different algorithms (LASTZ, LAGAN, MAVID and CONREAL). These factors include c‐MYB, E47, ETS, and GATA family members (Fig. 4A), which are expressed in the mammalian retina (Morrow et al. 1999; Crawford et al. 2002; Lee et al. 2004; Morris et al. 2011; Willardsen et al. 2014). Slightly further away, we also identified the conserved putative binding site for NeuroD that, together with its E47 cofactor, has been shown to transactivate the medaka enhancer region (Conte et al. 2010).

Taken together, these data support the idea that POAG patients carrying the identified 4 bp deletion might express lower SIX6 levels in the RGC and amacrine cells, which may predispose to develop POAG in adulthood.

## Discussion

POAG is a complex genetic disorder with heterogeneous clinical and genetic traits. As in the case of other disorders with complex inheritance pattern, the design of more effective treatments for POAG might benefit from understanding the specific association of genetic variants with given endophenotypes. In this study, we have thus aimed at determining the possible association of the *SIX6* gene and the *SIX1‐SIX6* locus with south Indian POAG cases, as well as with clinically relevant POAG parameters, such as RNFL and VCDR. Our results further implicate the *SIX1‐SIX6* locus and the *SIX6* gene with POAG and provide evidence for the association of two genetic risk variants, rs33912345 and rs10483245, with a significant increase in the VCDR in south Indian POAG cases. Besides being one of the first study of the south Indian population, our work identified a novel 4 bp deletion in a highly conserved *SIX6* retinal enhancer, which seems to impair its activity according to zebrafish transgenic assays. This suggests that changes of SIX6 expression in RGCs may predispose to the development of POAG in adulthood.

By sequencing the *SIX6* gene in a cohort of south Indian POAG cases and age‐matched controls, we identified both common and rare variants at different positions in the gene (Fig. 1A). Previous studies have shown that one of the common variant, rs33912345, that we have also identified, represents a POAG susceptibility factor in different ethnic populations (Osman et al. 2012; Carnes et al. 2014; Iglesias et al. 2014). In contrast with these studies, we could not establish significant association of this variant in the cohort we analyzed. Failure of finding such an association has been reported also for a cohort of POAG patients from Ghana, West African (Liu et al. 2013; Williams et al. 2015). Notably, in Ghana, the frequency of this allele in both the control and the POAG‐affected population reaches 99% (Williams et al. 2015). This may indicate that a very large fraction of both the south Indian and West African population is at high risk of developing POAG. Indeed, POAG prevalence in Ghana is the highest worldwide (Ntim‐Amponsah et al. 2004; Williams et al. 2015). Alternatively, other factors, as for example, additional variants in other genes or differences in the structure of the optic nerve may contribute to POAG onset in these regions.

An additional SNP, rs10483245, related to the SIX6 locus and positioned within 40 kb from the *SIX1/SIX6* gene cluster on Chr14q22‐23, has been previously associated with changes in VCDR (Ramdas et al. 2010; Fan et al. 2011; Mabuchi et al. 2012; Iglesias et al. 2014), increased IOP (Iglesias et al. 2014; Ozel et al. 2014) and POAG (Knowler et al. 2002; Ramdas et al. 2010, 2011; Khor et al. 2011; Osman et al. 2012). In our study, we did not reproduce the association of this SNP with our south Indian POAG cohort, supporting the conclusions of a previous study with a similar Indian population (Philomenadin et al. 2015). The reasons for the discrepancy between our study and that of Philomenadin et al. (2015) with those in other ethnical groups might be several, including the presence of additional risk factors in the Indian population, as mentioned above, or our sample size perhaps being not large enough to guarantee the detection of a true association.

Despite the lack of genetic association between the rs10483245 and rs33912345 SNPs and south Indian POAG, our study has identified a reduction in the global RNFL thickness in individual carrying the risk alleles (C and T) for either one of these variants (rs33912345 and rs10483245), as compared with POAG cases carrying nonrisk alleles. Although not statistically significant, likely because we are comparing variations among a limited number of POAG cases, this reduction was quite consistent and well in agreement with the results of the first study that established a correlation between a *SIX6* POAG risk allele (His141; associated with the common variant rs33912345) and a thinner RNFL (Carnes et al. 2014). A similar correlation was thereafter reported in studies of cohorts of European and Chinese POAG populations (Cheng et al. 2014; Kuo et al. 2015). As Carnes et al. (Carnes et al. 2014), we observed that the RNFL of POAG patients carrying the risk alleles was particularly thinner in the superior quadrant, which is known to affect directly the measurement of VCDR, a critical ocular biomarker for the diagnosis and progression of glaucoma (Ramdas et al. 2010). Consistently, we also identified a significant increase in the VCDR in POAG patients carrying the C and T risk alleles, providing further support to previous independent retrospective studies associating the *SIX1‐SIX6* locus and the *SIX6* gene with an increased VCDR (Ramdas et al. 2010; Fan et al. 2011; Mabuchi et al. 2012; Iglesias et al. 2014; Philomenadin et al. 2015). Taking all these data together, it seems reasonable to propose that the study of the association of *SIX1‐SIX6* and *SIX6* variants coupled with the measurement of the VCDR and RNFL thickness should be routinely used to screen individuals for the risk of developing glaucoma.

A particularly interesting aspect of our study is the identification of a novel 4 bp deletion within a previously described (Conte et al. 2010) and highly conserved *SIX6* enhancer. According to luciferase assays and the establishment of stable transgenic lines in zebrafish, this deletion highly impairs enhancer function. Notably, the human control enhancer drives expression in cells of the amacrine and RGC layers, coinciding with the reported expression of *SIX6* mRNA in human fetal retinas (Gallardo et al. 1999). This expression is completely absent or strongly reduced in transgenic lines carrying the deleted version of the enhancer found in POAG patients, strongly suggesting that these patients might have reduced levels of SIX6. This reduction, in turn, could result in less RGC number and thus in a thinner nerve fiber layer. Indeed, inactivation of *Six6* in both mouse and zebrafish causes optic nerve hypoplasia (Li et al. 2002; Iglesias et al. 2014). Furthermore, Carnes et al. (Carnes et al. 2014) have shown that this hypoplasia in zebrafish could be fully rescued by a *SIX6* human nonrisk allele but not by two risk alleles (Leu205Arg and Asn141His variants) found in POAG patients. *Six6* regulates retinal progenitors’ proliferation (Zuber et al. 1999; Li et al. 2002). Therefore, it is possible that lower level of SIX6 might slightly affect the generation of an appropriate number of RGCs, which might be in addition or alternatively more prone to death. This last possibility would be in apparent contrast with two recent studies suggesting that *SIX6* expression is instead upregulated in glaucoma. The first study found one variant of the retinal *SIX6* enhancer (Chr14:60974449_G) in POAG patients that resulted in a significant increase in reporter expression in luciferase assays as compared to the reference enhancer (Carnes et al. 2014). The second study showed that *Six6* expression is upregulated in a mouse model of acute glaucoma and that this upregulation directly increases the expression of p16INK4a (Skowronska‐Krawczyk et al. 2015), a cyclin‐dependent kinase inhibitor that is a hallmark of cellular senescence (Campisi 2013). This led to propose that increased *SIX6* expression would cause RGC senescence, a state that would culminate with cell death (Skowronska‐Krawczyk et al. 2015). The idea that both up‐ and down‐regulation of *SIX6* might cause glaucoma are however compatible, as SIX6 interacts with different cofactors, forming transcriptional activating and repressing complexes, thereby controlling different down‐stream targets (Yusuf et al. 2012), perhaps all involved in controlling RGC viability. Furthermore, haploinsufficiency or overexpression of the same gene can lead to similar phenotypes. Pax6, a transcription factor responsible for eye defects in mammals, is a striking example of the importance of correct gene dosage for eye formation (Schedl et al. 1996).

How the identified 4 bp deletion impairs enhancer function needs to be determined. However, we have identified binding sites for different factors in the enhancer sequence encompassing the deletion. These factors, including NeuroD, ETS, E47, GATA, and c‐MYB may account for enhancer regulation. NeuroD, in combination with E47, has been shown to activate this enhancer (Conte et al. 2010; Carnes et al. 2014) but its binding site is not affected by the deletion (Fig. 4A). c‐MYB, ETS, and GATA family members are more likely candidates. These factors are expressed in the vertebrate retina (Crawford et al. 2002; Lee et al. 2004; Morris et al. 2011; Willardsen et al. 2014) and, interestingly, ETS expression increases in the retina of a rat model for diabetes (Du et al. 2007), one of the factors that predispose to glaucoma. Nevertheless, we need to determine if their function is relevant to *SIX6* expression and, more importantly, if this deletion indeed predispose retinal ganglion cells to death, a possibility that could be verified by editing the zebrafish genome. These issues will be the objects of our future studies.
